# Characterising the Associated Virome and Microbiota of Asian Citrus Psyllid (*Diaphorina citri*) in Samoa

**DOI:** 10.3390/pathogens14080801

**Published:** 2025-08-10

**Authors:** Kayvan Etebari, Angelika M. Tugaga, Gayatri Divekar, Olo Aleni Uelese, Sharydia S. A. Tusa, Ellis Vaega, Helmy Sasulu, Loia Uini, Yuanhang Ren, Michael J. Furlong

**Affiliations:** 1 School of Agriculture and Food Sustainability, The University of Queensland, Brisbane 4072, Australia; 2 Scientific Research Organisation of Samoa (SROS), Apia P.O. Box 6597, Samoa; 3 Key Laboratory of Coarse Cereal Processing of Ministry of Agriculture and Rural Affairs, Chengdu University, Chengdu 610106, China; 4 School of the Environment, The University of Queensland, Brisbane 4072, Australia

**Keywords:** *Diaphorina citri*, insect-specific viruses, Samoan islands, symbiotic microorganisms

## Abstract

The Asian citrus psyllid (*Diaphorina citri*) is an economically important pest of citrus as it is a vector of the bacterium (*Candidatus* Liberibacter asiaticus, *C*Las) that causes huanglongbing disease (HLB). Understanding the virome of *D. citri* is important for uncovering factors that influence vector competence, to support biosecurity surveillance, and to identify candidate agents for biological control. Previous studies have identified several *D. citri*-associated viruses from various geographical populations of this pest. To further investigate virus diversity in this pest, high-throughput sequencing was used to analyse *D. citri* populations from the Samoan islands of Upolu and Savai’i. Eleven novel viruses from the *Yadokariviridae*, *Botourmiaviridae*, *Nodaviridae*, *Mymonaviridae*, *Partitiviridae*, *Totiviridae*, and *Polymycoviridae* were identified as well as some that corresponded to unclassified groups. In addition, microbiome analysis revealed the presence of several endosymbiotic microorganisms, including *Wolbachia*, as well as some plant pathogenic fungi, including *Botrytis cinerea*. However, the causative agent of HLB disease (*C*Las) was not detected in the RNA-Seq data. These findings highlight the complex and diverse microbiota associated with *D. citri* and suggest potential interactions and dynamics between microorganisms and psyllid-associated viruses. Further research is needed to understand the ecological significance of these discoveries, and whether the novel viruses play a role in regulating field populations of the psyllid.

## 1. Introduction

The Asian citrus psyllid (ACP, *Diaphorina citri*) is an extremely important vector of *Candidatus* Liberibacter asiaticus (*C*Las), the pathogen that causes huanglongbing (HLB), also known as citrus greening disease. HLB is one of the most serious diseases affecting citrus plants worldwide. HLB disrupts plant physiology by blocking phloem transport, causing chlorosis, root decline, and reduced photosynthesis, ultimately leading to tree decline. It has a significant negative economic impact on the citrus sector, particularly in major producing countries such as the United States, Brazil, and China. Globally, HLB is estimated to cause over 4.5 billion USD in economic losses annually [1,2,3,4]. Although HLB is not currently present in Australia, its occurrence in neighbouring countries and the presence of the vector pose a significant biosecurity threat to the Australian citrus industry. Therefore, understanding and managing the spread, dispersal, and population dynamics of *D. citri* is essential for effective HLB disease management and biosecurity surveillance in the region.

Historically, management efforts against ACP have relied primarily on chemical insecticides and antibiotics; however, these have shown limited long-term efficacy, with increasing reports of insecticide resistance in ACP populations and inconsistent disease suppression, particularly under field conditions [5]. For example, resistance to neonicotinoids and pyrethroids has been documented in ACP populations in regions such as Florida and Brazil, significantly reducing control effectiveness [6,7]. In addition, the extensive use of chemical controls has contributed to environmental contamination and has had detrimental effects on non-target and beneficial organisms within citrus orchard. The constraints of chemical control have generated interest in developing more sustainable, environmentally friendly approaches to managing *D. citri* populations and reducing the transmission of HLB.

As a result, research has expanded beyond traditional biological control to investigate the potential of various microorganisms that can alter *D. citri* fitness and interfere with disease transmission. This includes the use of entomopathogenic fungi, bacteria, viruses, and protozoa, which have shown promise in weakening vector populations or altering their capacity to spread the pathogen. For instance, a study in China has demonstrated that endosymbiotic viruses isolated from *D. citri* can significantly reduce egg production in female psyllids, thereby limiting population growth [8].

Building on this, metagenomic analysis has revealed the diversity and abundance of both DNA and RNA viruses in insects, shedding light on the viral communities associated with vectors like *D. citri*. Several viruses have been identified in *D. citri*, and these can be grouped based on their genome types: DNA viruses such as Diaphorina citri densovirus (DcDV) and densovirus-like sequences and RNA viruses including reovirus (DcRV), bunyavirus (DcBV), flavi-like virus (DcFLV), associated C virus (DcACV), and picorna-like viruses (DcPLV). In addition, several unclassified positive-sense single-stranded RNA viruses have also been discovered, but their taxonomic placement remains unresolved, and their biological roles in *D. citri* are still unknown. [9,10].

Due to their high adaptability to insect hosts, psyllid-specific viruses have the potential to interfere with the transmission of insect-borne pathogens responsible for diseases such as HLB, citrus tristeza, and citrus yellow vein disease [11]. However, recent findings indicate a more complex interaction. For example, the presence of DcFLV has been shown to enhance the transmission of *C*Las by both nymphs and adults. DcFLV appears to modulate various cellular and physiological processes in *D. citri* in a life stage-dependent manner, promoting *C*Las acquisition during the nymphal stage and facilitating transmission in adults. In nymphs, DcFLV suppresses defence and autophagy genes and may enhance viral movement via vesicular transport, facilitating higher *C*Las acquisition. In adults, DcFLV induces ER stress and immune responses, leading to cell damage and reduced *C*Las acquisition, but prolonged infection may still support transmission by increasing bacterial titre. These findings suggest that *D. citri* individuals infected with DcFLV may be more efficient vectors of *C*Las compared to virus-free counterparts [10].

In addition to influencing pathogen transmission, these viruses can alter host traits and physiological processes. For example, virus-induced gene silencing (VIGS) can be used to target specific insect RNAs, or some viruses may stimulate host immune responses against other pathogens [12,13]. Despite these advances, the diversity of unidentified viruses in psyllid populations and the mechanisms by which *D. citri*-associated viruses influence host physiology and pathogen transmission remain poorly understood. Further research is needed to characterise these interactions and their implications for disease ecology and pest management. Identifying virus-specific gene targets involved in immune modulation, or determining how co-infection alters vector competence and pathogen load, can inform disease ecology and pest management strategies.

In this study, we employed an RNA-Seq approach to investigate the diversity of viruses in *D. citri* collected from the two Samoan islands, Upolu and Savai’i, between 2019 and 2022. Samoa was selected for this study due to the widespread presence of *D. citri* and the current absence of HLB, providing a valuable setting to characterise the baseline virome and microbiota. This research primarily strengthens Samoa’s preparedness by establishing foundational knowledge to support early detection, future surveillance, and pest management strategies. It also contributes to regional citrus biosecurity efforts in neighbouring countries such as Australia and New Zealand. 

The near-complete genomes of eleven novel viruses were identified, along with traces of several bacterial species and some plant-pathogenic fungi in different psyllid populations. Genomic profiling of insect vectors, or Vector-Enabled Metagenomics (VEM) survey, offers a powerful biosecurity tool for *D. citri* surveillance by enabling the early detection of diverse and potentially emergent plant viruses carried by the vector, even before symptoms appear in crops [14,15,16]. This proactive approach enhances our capacity to monitor viral threats and manage plant disease risks more effectively

The findings will highlight the presence of a complex and diverse microbial community associated with *D. citri*, including insect-specific viruses and potential endosymbiotic bacteria. While the functional roles of these microorganisms remain to be explored, their detection provides a foundational resource for future studies on microbe-insect-pathogen interactions and their possible applications in managing psyllid populations and limiting the spread of HLB disease.

## 2. Materials and Methods

### 2.1. Survey and Insect Collection

*Diaphorina citri* adult were collected from various locations across the islands of Upolu (21 sites) and Savai’i (20 sites) in Samoa between 2019 and 2022. Approximately 437 adult psyllids were randomly sampled from multiple trees by using an insect aspirator (Supplementary Figure S1). Adult psyllids from each sampling site were preserved in ethanol or an RNA stabilization reagent (RNAprotect^®^, QIAGEN Cat No.: 76104) for downstream DNA and RNA extraction and sequencing. RNAprotect was used for transcriptomic analysis, while ethanol-preserved samples were retained for DNA-based assessments or backup purposes.

### 2.2. Conventional PCR Approach for Host Identification and Pathogen Detection

DNA from 287 samples was extracted by DNeasy Blood and Tissue Kits for DNA Isolation (QIAGEN Cat No.: 69504). DNA quantity and quality were assessed using a NanoDrop spectrophotometer. All extracted samples were screened for the presence of *C*Las by using polymerase chain reaction (PCR) targeting the 16S rRNA gene, a conserved region commonly used in bacterial identification and known for its specificity in detecting *C*Las [17]. All PCRs were run in duplicate with positive and negative controls to ensure proper amplification, reproducibility, and absence of contamination. To screen for the presence of bacteria in the samples, we used primers considered diagnostic for *C*Las. The primer sequences were HLB75F (5′–CGCGTATGCAATACGAGCGGCA–3′) and HLB177T R (5′–GCCTCGCGACTTCGCAACCCAT–3′) [17].

### 2.3. RNA Extraction and Sequencing

A total of 150 adult *D. citri* collected from Upolu and Savai’i in 2022 was divided into three groups for RNA-Seq analysis. One group consisted of samples from Savai’i, while the other two groups comprised samples collected from the northern and southern regions of Upolu. Savai’i (SAV) was treated as a single group because the sampling sites on this island were in close proximity to each other, limiting spatial variability. In contrast, Upolu has a wider geographic spread with more dispersed citrus trees, so it was divided into northern (UPO1) and southern (UPO2) regions to better reflect potential regional differences in virome and microbiota composition.

Adult insects were transferred into QIAzol lysis reagent, and total RNA was extracted following the manufacturer’s protocol (QIAGEN; Cat No.: 79306). Briefly, insects were homogenised in QIAzol and incubated at room temperature, and phase separation was achieved by adding chloroform and centrifugation. The aqueous phase was transferred, and RNA was precipitated using isopropanol, washed with 75% ethanol, and air-dried. The RNA pellet was finally resuspended in RNase-free water for downstream applications.

The RNA samples were treated with DNase I for 1 h at 37 °C. Concentrations were measured using a spectrophotometer, and integrity was confirmed by electrophoresis on a 1% (*w*/*v*) agarose gel. After verifying RNA quality, total RNA from three pooled groups (SAV, UPO1, and UPO2) was submitted to the Novogene sequencing facility in Hong Kong for library preparation (following eukaryotic ribosomal RNA depletion) and strand-specific total RNA sequencing on the NovaSeq platform. The eukaryote ribosomal RNA was removed using a VAHTS Total RNA-seq (HMR) Library Prep Kit for Illumina. 

### 2.4. Bioinformatics Analysis and Virus Discovery

The CLC Genomics Workbench version 21.0.5 and OmicsBox 3.0.25 were used for bioinformatics analyses. All sequencing libraries were trimmed to remove residual adapter or vector sequences. Reads with a quality score below 0.05 and those containing more than two ambiguous nucleotides were excluded from further analysis. Quality filtering resulted in minimal read loss, with 0.83% for UPO1, 0.99% for UPO2, and 1.57% for SAV libraries. The majority of high-quality reads were retained, with an average read length of 148 base pairs used for downstream analysis. All trimmed reads were mapped to the *D. citri* genome (GCA_000475195.1) to remove host-related sequences. The unmapped reads were retained for downstream analysis in our virus discovery pipelines or microbiome study by Kraken2 tools in OmicsBox. We used meta-SPAdes [18] with automatic k-mer size for de novo assembly in the metagenomic mode after removing rRNA with SortMeRNA [19]. We also used another de novo assembly approach in CLC (word size 25, bubble size 50, and minimum contig length 300 bp) to process these data and verify the best assembly outcomes. The contigs were corrected by mapping all reads against the assembled sequences (min. length fraction, maximum mismatch, insertion, and deletion cost of 0.8, 2, 3, and 3 respectively). Assemblies were compared based on N50, total contig length, and contig count > 1 kb. Meta-SPAdes outperformed CLC and was selected for downstream analysis.

For virus discovery, assembled contigs were initially compared to locally curated NCBI viral genome and protein databases (downloaded December 2024) using BLASTn and BLASTx, applying a stringent e-value cutoff of 1 × 10^−10^ to enhance sensitivity and reduce false positives. Contigs with viral hits were further validated using BLASTx against the non-redundant protein sequences (nr V5) database via OmicsBox to confirm viral origin and eliminate spurious matches. To detect highly divergent viruses, we also performed domain-based searches by comparing the assembled contigs against the Conserved Domain Database (CDD) version 3.14 and Pfam v32 with an expected value threshold of 1 × 10^−3^. Sequences with positive hits to virus polymerase (RNA-dependent RNA polymerase (RdRP) domain: cd01699) were retained. Viral contigs were further validated by cross-checking ORF integrity and alignment with known viral genomes across both assemblies.

To explore the microbial community of ACP, the paired-end total RNA reads were used as input in Kraken 2, which is a taxonomic sequence classifier that assigns taxonomic labels to short reads [20]. We applied a Kraken confidence filter of 0.05 and a minimum hit group of 5 for a k-mers-based querying against the RefSeq WGS database (version 2024–11) database including archaea, bacteria, fungi, protozoa, and viruses. This database maps each k-mer to the lowest common ancestor (LCA) across genomes in the taxonomic tree. To reduce false positives and laboratory artifacts, raw reads were first mapped against known contaminants, including the host insect genome and PhiX, using Bowtie2. Only unaligned reads were retained for microbial profiling. The set of LCA taxa that correspond to the k-mers in a read are then analyzed to create a single taxonomic label for the read [21].

### 2.5. Phylogenetic Analysis of Identified Viruses

The deduced amino acid sequences of predicted ORF regions or RdRP of newly identified viruses were used to calculate their phylogenetic relationship with other members of each respective family. Closely related viruses were identified through BLASTp analysis against the NCBI non-redundant protein database (nr V5). Viral protein sequences with ≥30% amino acid identity and ≥50% query coverage were downloaded and used for downstream phylogenetic analysis and comparison.

Multiple amino acid sequence alignments were generated using MUSCLE. Phylogenetic trees were inferred using the IQ-TREE web server (ModelFinder v2.1) to identify the best-fit substitution model based on Bayesian Information Criterion (BIC). We used the JTT+G model with discretised gamma rate distribution (four categories), a gamma shape parameter of 1.0, and 1000 bootstrap replicates. Appropriate outgroups were selected for each phylogenetic tree.

## 3. Results and Discussion

High-throughput sequencing of the *D. citri* RNA samples from Savai’i (SAV), northern Upolu (UP01), and southern Upolu (UP02) generated 90,858,236; 98,811,204; and 121,917,432 raw reads for each sample, respectively. On average, 80–85% of reads mapped to the *D. citri* genome and were excluded from downstream viral analysis. Assembly using MetaSPAdes produced an average of 44,382 scaffolds longer than 500 base pairs. These assembled sequences were then queried against the NCBI database using BLAST, revealing similarity to a range of insect-specific and plant viruses present in the gut samples from all these populations. Several novel viral sequences (summarised in Table 1 and Table 2) were identified. However, due to the lack of definitive evidence confirming *D. citri* as the host organism, we refer to them as Diaphorina citri-associated viruses.

### 3.1. Single-Stranded Positive-Sense RNA Viruses

#### 3.1.1. Hypoviridae

**Figure 1:**
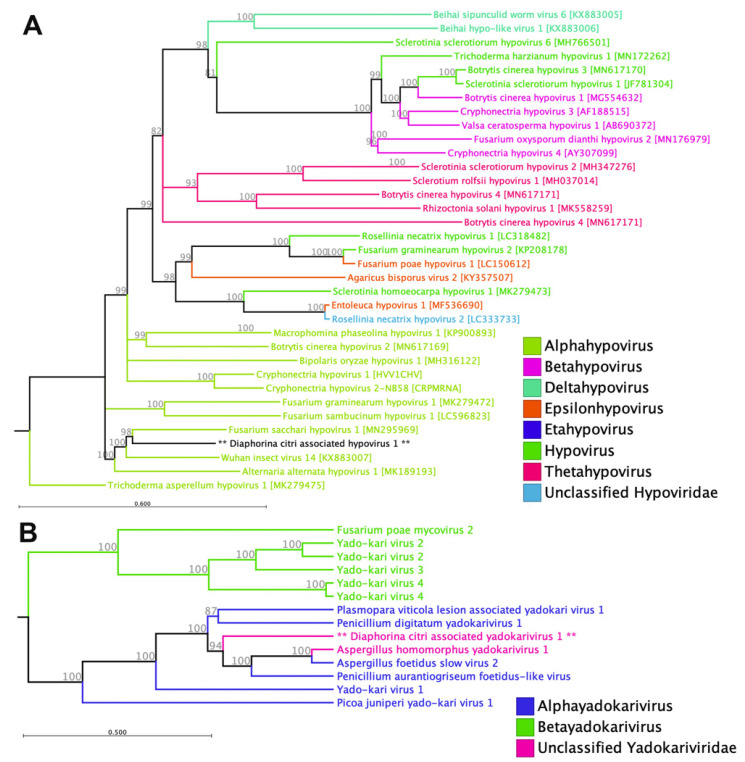
Phylogenetic relationships of putative single-stranded positive-sense RNA viruses detected in *D. citri*. (**A**) *Hypoviridae*. (**B**) *Yadokariviridae*. Novel viruses identified in this study are indicated with an asterisk (**).

We identified a new member of *Hypoviridae* from *D. citri* samples collected from Samoa and tentatively named it Diaphorina citri-associated hypovirus 1 (DcHV1). This sequence is accessible with NCBI GenBank accession number of PV821379. Members of this family are capsid-less viruses with positive-sense RNA genomes of 7.3–18.3 kb that possess either a single large open reading frame (ORF) or two ORFs [22]. The complete genome sequence consisted of a single positive-stranded RNA genome of 11,431 nucleotides and included one open reading frame with 3468 amino acids and a predicted molecular weight of 394.437 kDa (Table 1). We detected four major domains including DNA/RNA polymerase, P-loop containing nucleoside triphosphate hydrolase, Helicase superfamily (ATP-binding domain), and a protein domain of unknown function (DUF3525) on this viral sequence (Table 2). The new virus (DcHV1) was exclusively detected in *D. citri* samples obtained from the northern region of Upolo, with no evidence of its presence found in any other areas. Based on multiple alignments of the deduced amino acid sequences of their predicted polyproteins, phylogenetic analysis showed that DcHV1 and Wuhan insect virus 14, which is an unclassified hypovirus identified in fleas and ants through meta-transcriptomics, were placed under the Alphahypovirus clade along with other hypoviruses isolated from fungi (Figure 1A).

DcHV1 exhibited the highest level of sequence similarity at the amino acid level with Chuzhou tick virus 1 (UYL95331.1), which was previously detected in the Asian monitor lizard tick (*Amblyomma varanense*). The recently identified viral genome exhibited amino acid sequence identities of 57.73% and 57.11% with Wuhan insect virus 14 and Apis hypovirus 1, respectively. Additionally, it shared 57.29% sequence identity with Fusarium sacchari hypovirus 1, which was identified in *Fusarium sacchari* strain FJ-FZ06. This strain is known to be a causative agent of sugarcane Pokkah Boeng disease in China [23]. Filamentous fungi from both the ascomycetous and basidiomycetous groups have been found to harbor hypovirids. These hypovirids replicate within lipid vesicles derived from the host’s Golgi body and contain double-stranded RNA (dsRNA) as their replicative form [22]. One well-studied member of the *Hypoviridae* is Cryphonectria hypovirus 1 (CHV1/EP713), which has proven to be an effective biological control agent against the chestnut blight pathogen *Cryphonectria parasitica* [24]. There have been recent reports of members of this family in arthropods [25]. However, additional experimental validations are required to confirm these arthropods as true hosts.

#### 3.1.2. Yadokariviridae

The *Yadokariviridae* is currently the sole family in the order Yadokarivirales, which was established in 2021 [26]. This family comprises two genera, Alphayadokarivirus and Betayadokarivirus, and consists of non-segmented positive-sense (+) RNA viruses without a capsid. These recently proposed family members are all fungal viruses, with genome sizes ranging from 3.6 to 6.3 kb and encoding either one or two open reading frames. We identified a single-segment genome sequence of a putative virus (GenBank accession number: PV821380) from the newly established family *Yadokariviridae* and tentatively named it Diaphorina citri-associated yadokarivirus 1 (DcYv1). The RNA genome consists of 3828 nucleotides, including one open reading frame with 984 amino acids and a predicted molecular weight of 113.104 kDa with an isoelectric point of 9.79 (Table 1). BLASTp searches revealed that the deduced amino acid sequence of its predicted ORF has the highest shared identity (58.68%) with the polyprotein Aspergillus homomorphus yadokarivirus 1 (AZT88626.1), which has previously been reported from the USA [27]. The complete polyprotein of this novel virus also showed around 57% shared identity with 92% coverage with RdRp of Aspergillus foetidus slow virus 2. The RNA-dependent RNA polymerase (RdRP) of yadokarivirids exhibits similarities to various groups of positive-strand RNA viruses within the phylum Pisuviricota. Although yadokarivirids are distantly related to calicivirids that infect animals in the order Picornavirales, they differ in terms of genome organization. We only identified a major domain of RNA-directed RNA polymerase (C-terminal domain) on the viral polyprotein. This viral genome sequence was assembled from 15,746 reads and has only been identified in samples collected from the north of Upolo (Table 2). The maximum likelihood phylogeny analysis placed this novel virus within the Alphayadokarivirus group, alongside other fungal viruses from this family (Figure 1B).

*Yadokariviridae* viruses have been discovered in various ascomycetous fungi and environmental samples worldwide [27]. It has been observed that all known yadokarivirids coexist with diverse dsRNA viruses within a single host fungus, indicating a potential partnership [26]. However, during our investigation, we only identified one contig exhibiting sequence similarity to known yadoka-like viruses. While each yadokarivirid demonstrates species-level specificity with its partner, as a whole, yadokarivirids have the ability to associate with diverse dsRNA viruses from two families, one genus that has yet to be assigned to a family, and two additional unclassified groups within the *Ghabrivirales* order. To the best of our knowledge, there are no reports of this virus infecting insects or other arthropods, and it is more likely that we identified DcYv1 in some fungi-infected *D. citri*. Nonetheless, further research is necessary to comprehensively understand the diversity, ecology, and biological significance of *Yadokariviridae*.

#### 3.1.3. Botourmiaviridae

The family *Botourmiaviridae* includes mono- or multi-segmented viruses with positive-sense RNA genomes that mainly infect plants and fungi. It consists of twelve genera, among which *Ourmiavirus* is notable as it represents plant viruses with non-enveloped bacilliform virions composed of a single coat protein. Additionally, *Botoulivirus*, *Magoulivirus*, and *Scleroulivirus* are the primary genera known for infecting fungi. These viruses have genomes ranging in size from 2 to 5 kb, containing a single open reading frame [28]. Recently several new members of this family have been identified in metagenomic analyses of insect and tick viriomes [29,30]. We identified two viral sequences similar to previously reported viruses from *Botourmiaviridae* and tentatively named them Diaphorina citri-associated ourmia-like viruses 1 and 2 (DcOv1 and 2) mainly from Savai’i. Each RNA genome consists of 1739 (DcOv1) and 1134 (DcOv2) nucleotides, including one open reading frame with 495 and 375 amino acids, respectively. The newly identified sequences were deposited in the NCBI GenBank under the accession numbers of PV821381 and PV821382.

**Figure 2:**
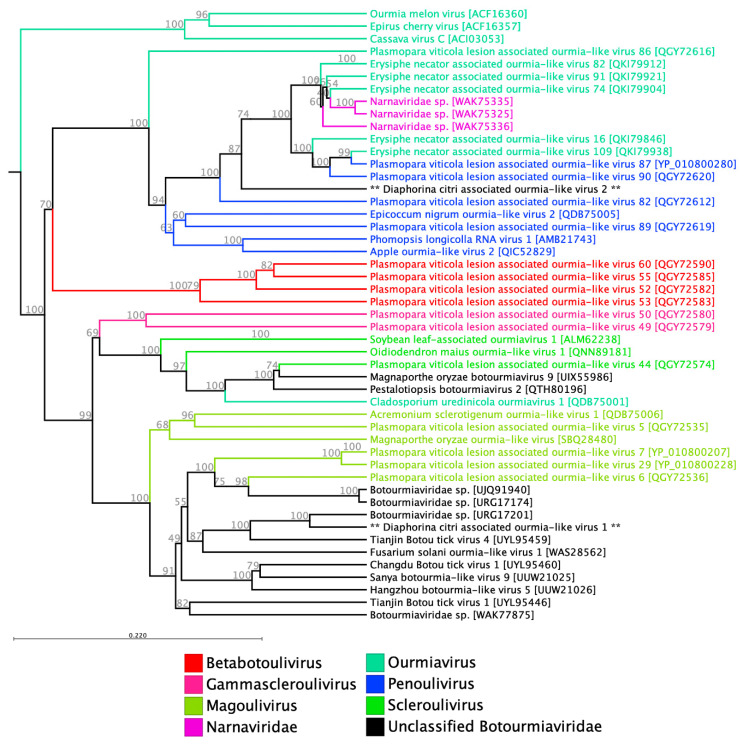
Phylogenetic relationships of two putative *Botourmiaviridae* members detected in *D. citri*, tentatively named Diaphorina citri-associated ourmia-like viruses 1 and 2 (DcOv1: PV821381 and DcOv2: PV821382). Novel viruses identified in this study are indicated with an asterisk (**).

The DNA/RNA polymerase superfamily domain has been detected on the DcOv1 sequence, while no conserved domain has been identified on DcOv2. The deduced amino acid sequence of DcOv1 shows 50.51% shared identity with Tianjin Botou tick virus 4, which was recently reported from *Haemaphysalis longicornis* ticks in China. Moreover, DcOv1 clusters with other members of this family, including Fusarium solani ourmia-like virus 1, a fungal virus, with 47.76% shared identity. DcOv2 displayed a 53% sequence similarity to another recently identified virus found in the same tick species (*H. longicornis*). Furthermore, DcOv2 exhibited significant similarity to other ourmia-like viruses isolated from the grape powdery mildew fungus (*Erysiphe necator*). These related viruses include *Erysiphe necator*-associated ourmia-like virus 16, 109, 82, 91, and 74, which all are assigned to the genus Ourmiavirus (Figure 2).

Leviviridae-like viruses, including *Botourmiaviridae*, *Narnaviridae*, and *Mitoviridae*, share close evolutionary relationships and have diverged from Lenarviricota, the direct descendant of bacteriophages. Recent studies have identified additional members of these virus families in invertebrates such as insects and ticks [30]. It has been proposed that horizontal virus transfer among different hosts, including plants, fungi, and invertebrates, played a significant role in shaping the evolutionary pathway of Lenarviricota [31]. Recently, a meta-analysis revealed the discovery of two new members of the *Botourmiaviridae* family from publicly available RNA-Seq data of a cockroach (*Loboptera decipiens*) and a euonymus scale insect (*Unaspis euonymi*) [29]. Furthermore, approximately 25% of the total newly identified viruses in a recent metavirome analysis of 31 tick species were classified under *Botourmiaviridae*, with reads associated with this viral family being highly abundant across most examined tick genera [30].

#### 3.1.4. Other ssRNA (+) Viruses

**Figure 3:**
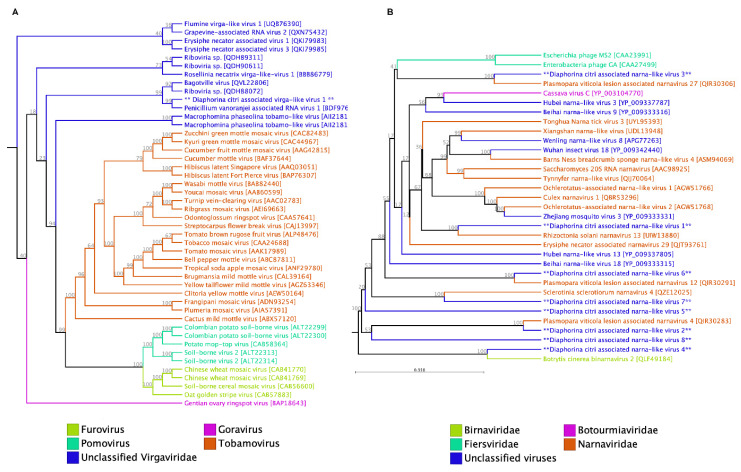
Phylogenetic relationships of putative single-stranded positive-sense RNA viruses from the families *Virgaviridae* (**A**) and *Narnaviridae* (**B**) detected in *D. citri*. Novel viruses identified in this study are indicated with an asterisk (**).

We identified a 1650 nt viral sequence with a single open reading frame, which contains C-terminal and catalytic domains of RdRP in the family *Nodaviridae* of positive-sense single-stranded RNA viruses. The *Nodaviridae* contains two genera that infect insects (*Alphanodavirus*) or fish (*Betanodavirus*). An important example of a virus from this family is the Nodamura virus, which was initially isolated from *Culex tritaeniorhynchus* mosquitoes in Japan [32]. Interestingly, despite possessing a conserved *Nodaviridae* RdRp domain, this newly identified virus associated with *D. citri* does not exhibit any similarity to other members of this family. Instead, a BLASTx analysis revealed its similarity to Penicillium vanoranjei-associated RNA virus 1, along with other virga-like and unclassified viruses. However, the phylogenetic analysis did not associate it with any other defined genus in the *Virgaviridae* family but rather grouped it with some other unclassified virga-like viruses that were previously reported from fungi (Figure 3A).

The *Virgaviridae* family includes plant viruses characterized by their rod-shaped virions and a single-stranded RNA genome. Within this family, the typical member is the Tobacco mosaic virus, which belongs to the *Tobamovirus* genus. Currently, there are seven approved genera with approximately 60 species classified under this family. The genome size of these viruses ranges from 6.3 to 13 kilobases (kb) of positive-sense RNA [33]. In the *Tobamovirus* genus, the genome is non-segmented, while in other genera, it is multipartite, with segments separately encapsidated in two or three components. In recent metagenomic studies, numerous novel insect-associated viruses closely related to members of this family have been reported [34,35]. There is also a possibility that this sequence has become integrated into the insect genome, justifying its classification as an endogenous viral element (EVE). In a previous study, several viral-like fragments exhibiting similarity to virga-like viruses were annotated as EVEs in neotropical *Mansoniini* mosquito species [36]. In our study, the viral fragment identified was smaller than the expected size range for members of the *Virgaviridae* family, and only a limited number of reads corresponding to this virus were detected in a single sequenced sample from individuals collected on Savai’i. This sequence (DcV1) has been submitted to NCBI GenBank under the accession number PV821383 (Table 2).

We also identified several small narna-like viruses in *D. citri* transcriptome data (DcNV1-DcNV10). The newly identified sequences were deposited in the NCBI GenBank under the accession numbers PV821384 to PV821393 (Figure 3B). The members of the family *Narnaviridae* do not encode capsid proteins, and their genomes are a single positive-strand RNA molecule ranging from about 2.3 to 3.6 kb [37]. This family includes two genera, *Narnavirus* and *Mitovirus*, whose members have a similar genome organization but differ in their subcellular localization, and they have been found in cytosol and mitochondria, respectively [37]. The DcNV3 possesses the longest viral genome, spanning 3552 nucleotides (nt), and exhibits a significantly larger assembly coverage (1126.5×). This particular narnavirus was consistently detected across all RNA-Seq libraries from both Samoan islands. Notably, a large number of reads corresponding to DcNV9 were observed in all geographic samples. In contrast, other members of this virus family were only detected in samples collected from the northern region of Upolu (Table 2).

Although the members of the *Narnaviridae* family have traditionally been identified as associated with plant, fungi, and protozoa [38,39,40,41], several recent studies have found many narna-like viruses in invertebrates including insects [42,43]. As ACP can potentially carry a variety of fungi and bacteria, we can assume that DcNV possibly replicates in insect-associated yeast or insect endosymbiont microbiota. However, a recent study showed that a novel narnavirus (CxNV1) can replicate in a *Culex tarsalis* cell line that is free from any fungal or bacterial contamination [42]. Another recent study identified several members of *Narnaviridae* in ectoparasitic fleas (*Spilopsyllus cuniculi* and *Xenopsylla cunicularis*) and their associated vertebrate host, the European rabbit (*Oryctolagus cuniculus*) [43]. Narnaviruses have a single ORF that encodes viral RNA-dependent RNA polymerase; however, another ORF has been determined on the minus strand of the closely related narnavirus in *Culex* (CxNV1) with no homology to other known viral proteins [42]. In some of these novel narna-like viruses, we also identified a smaller ORF in the negative strand of the viral genome, which can perhaps facilitate viral infection in insects.

**Figure 4:**
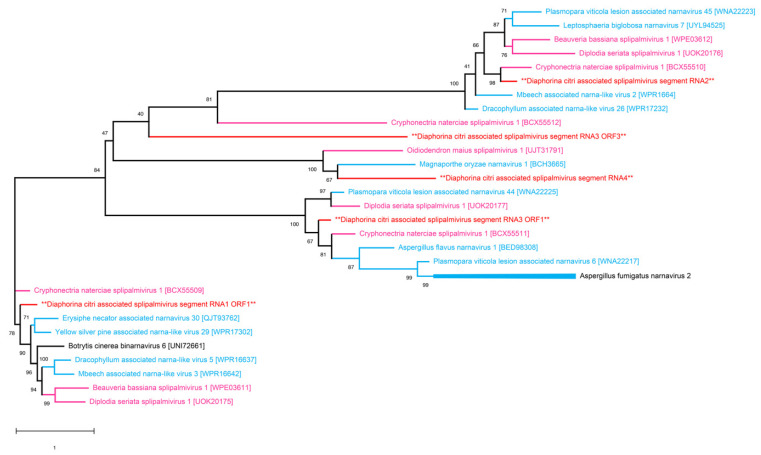
Maximum-likelihood phylogenetic tree of Diaphorina citri-associated splipalmivirus (DcSV1) segments, based on amino acid sequences of predicted ORFs. DcSV1 RNA segments 1–4 are marked with asterisks (**) and cluster with fungal splipalmiviruses, particularly Cryphonectria naterciae splipalmivirus 1, suggesting a likely fungal origin. Colour code: blue—*Narnaviruses*; pink—*Splipalmiviruses*; red—viruses identified in this study.

Splipalmiviruses are recently classified viruses that have a distinct characteristic of splitting the RdRp palm domain into two proteins [44]. They are similar to yeast narnaviruses and belong to the phylum *Lenarviricota* and the kingdom *Orthornavirae*. The genome is comprised of multi-segmented, positive-sense, single-stranded RNA [45]. We identified four RNA segments of a novel *Splipalmivirus*, temporarily named Diaphorina citri-associated splipalmivirus (DcSV1). The corresponding sequences are available in the NCBI GenBank under accession codes PV821394–PV821397. The majority of the segments were isolated from the *D. citri* population collected in northern Upolo, with only three read counts in the southern Upolo sample and none isolated from the Savai’i sample. RNA1 and RNA2 segments of DcSV1 had genome lengths of 2164 and 2273 nucleotides, respectively. The RNA3 segment was 1459 nucleotides long, while the RNA 4 segment was significantly shorter with only 533 nucleotides. Two of the segments, RNA2 and RNA4, showed a single open reading frame, while RNA1 had two open reading frames. RNA3 had the most ORFs with four ORFs. No identifiable domains were observed for the novel DcSV1. The BLASTp search of the RNA1 segment of the novel virus showed sequence similarity of 70.10% with RNA-dependent RNA polymerase of Cryphonectria naterciae splipalmivirus 1 (BCX55509.1). The RNA 2 and RNA 3 segments also showed maximum sequence identity with hypothetical proteins of this same virus. In the phylogenetic analysis, the ML tree also showed close grouping of segments 1, 2, and 3 with Cryphonectria naterciae splipalmivirus 1 (Figure 4). As this is a fungal virus, it suggests that *D. citri* is not the primary host of the novel DcSV1.

### 3.2. Single-Stranded Negative-Sense RNA

#### 3.2.1. Mymonaviridae

We identified a member of the mymona-like virus in all three RNA-seq libraries, with an average assembly coverage of 92.17x. The putative viral sequence, spanning 9708 nucleotides, was assembled from 9730 Illumina short reads. This viral sequence showed the highest abundance in the *D. citri* population in northern Upolo, with 5353 reads, and a lower density in the sample collected from Savai’i, with 1261 reads. We provisionally named this virus Diaphorina citri-associated mymona-like virus 1 (DcMv1), and the sequence has been submitted to NCBI GenBank under the accession number PV821398. The DcMv1 genome contains five major non-overlapping open reading frames, which encode proteins referred to as p I (415 aa), NP (269 aa), p III (290 aa), p IV (143 aa), and L (1910 aa) protein. In our analysis of the viral genome sequence, we identified two significant domains: the *Mononegavirales* RNA-directed RNA polymerase catalytic domain and the *Mononegavirales* mRNA-capping domain V (Table 2). These domains play crucial roles in the replication and transcription of the viral RNA. The discovery of DcMv1 and the characterization of its genome provide valuable insights into the genetic composition and diversity of mymona-like viruses.

**Figure 5:**
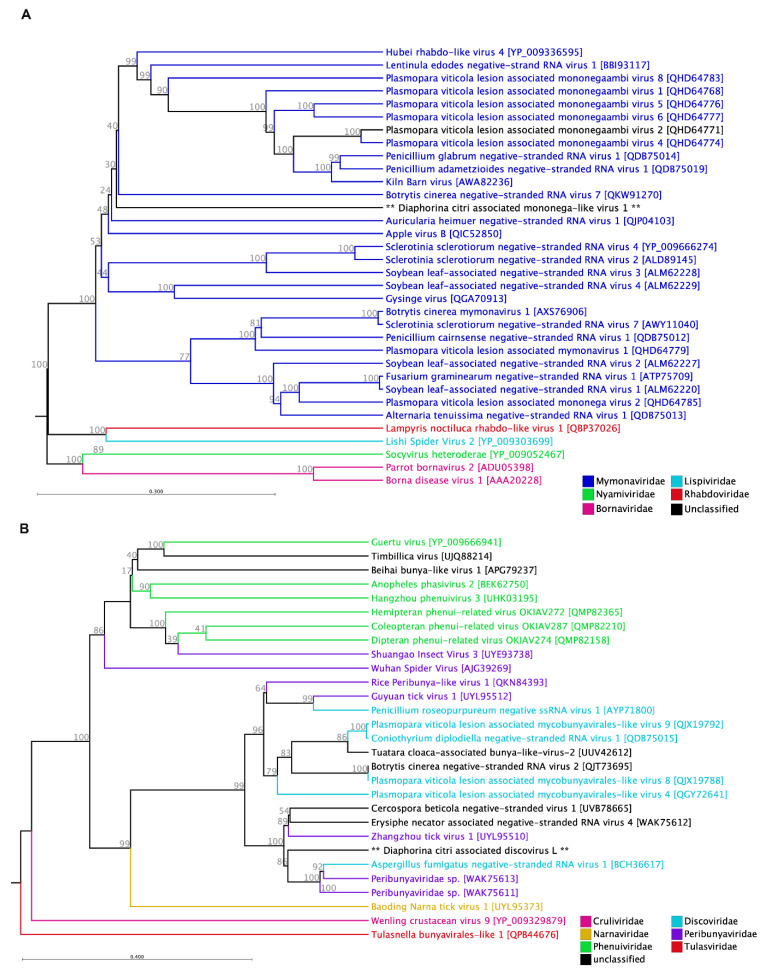
Maximum-likelihood phylogenetic tree of putative single-stranded negative-sense RNA viruses detected in *D. citri*. Phylogenetic relationships of Diaphorina citri-associated mymona-like virus 1 with other members of the family *Mymonaviridae* (**A**), and Diaphorina citri-associated discovirus segment L with other members of the family *Discoviridae* (**B**). Novel viruses identified in this study are marked with asterisks (**).

The L protein of DcMv1 exhibits a sequence identity of 57.60% with 26% coverage of *Leptosphaeria biglobosa* negative single-stranded RNA virus 4 (Table 1). This particular virus was identified through a metagenomic study of environmental samples in China. In addition, closely related viruses have been discovered in various invertebrate samples. Hubei rhabdo-like virus 4 (YP_009336595.1) was previously detected in a mixture of arthropod samples in China, Kiln Barn virus (YP_010797581.1) was isolated from *Drosophila suzukii* in the UK, and Jimsystermes virus (YP_010800904.1) was found in Australian termites (*Occasitermes* sp.). The L protein of DcMv1 shares sequence identities of 30.94%, 31.26%, and 29.38% with these viruses, respectively. Based on our phylogenetic analysis and examination of the genome structure, it is suggested that DcMv1 potentially belongs to the genera *Hubramonavirus* and is distinct from other members of the *Mymonaviridae* family (Figure 5A).

In this study, the newly discovered virus DcMv1was identified belonging to *Mymonaviridae*, a family of negative-strand RNA viruses within the order Mononegavirales, with fungi serving as their natural hosts. While mymonavirids typically infect filamentous fungi, there have been a few instances where they have been identified in association with insects, oomycetes, or plants [46]. In the previous meta-analysis conducted by Wu et al. (2020), it was observed that the mononega-like viruses were the predominant single-stranded RNA (ssRNA) viruses identified, being present in over 15% of the sampled insect species [29]. These findings highlight the high prevalence and importance of mononega-like viruses within insect populations. These viruses are characterized by the production of filamentous, enveloped virions containing a single molecule of linear RNA, typically measuring around 10 kilobases in length. Within the *Mymonaviridae* family, there are nine genera and nearly 50 known species. One well-studied virus in this family is Sclerotinia sclerotiorum negative-stranded RNA virus 1, which exhibits distinctive genomic features such as the absence of a poly(A) tail at the 3′-terminus and an uncapped 5′-terminus. Further investigation is required to assess the impact of DcMv1 on the *D. citri* population and its role in the ecology and dynamics of viral infections within this species. While this family is primarily known for infecting fungi, the identification of other members of this family in arthropods, detection of DcMv1 in all three *D. citri* RNA-Seq libraries, and with its high coverage raise the possibility that these viruses may also infect insects. In some cases, insects can serve as vectors for fungal viruses, facilitating their distribution. Previous reports have indicated that fungal viruses, such as Sclerotinia sclerotiorum hypovirulence-associated DNA virus 1, can be transmitted through insect vectors, expanding our understanding of the transmission mechanisms of fungal viruses beyond traditional routes [47]. This highlights the need for comprehensive studies to explore the potential role of insects as vectors for the dissemination of mycoviruses, including DcMv1.

#### 3.2.2. Discoviridae

We have discovered a new member of the recently proposed *Discoviridae* family (Figure 5B). The member of this family, a negative-sense bunyavirus-like RNA virus, was originally identified in *Penicillium roseopurpureum* during a virome study of fungi associated with the sea cucumber *Holothuria poli* [48]. Based on the genome organization and phylogenetic analysis of the viral RdRp, it is evident that this virus shares similarities with bunyaviruses but may not fit precisely within the established taxa or specific subgroups of the bunyavirus family. Therefore, in 2021, the proposal was made to establish a new genus called *Orthodiscovirus* within the newly proposed family, *Discoviridae* [49]. This virus consists of three monocistronic genome segments, which encode different components: a RdRp (RNA1), a non-structural protein (Ns; RNA2), and a nucleocapsid (Nc; RNA3). Among these segments, the longest one (DcDv-L) is 6556 nucleotides long and encodes a single open reading frame consisting of 2131 amino acids. The other two segments, M and S, are shorter in length, with 1221 and 1054 nucleotides, respectively, and each encodes a polyprotein. The newly identified sequences were deposited in the NCBI GenBank under the accession numbers of PV821414, PV821415, and PV821416. The bunyaviral RdRp domain has been detected within the L segment of this particular virus. The deduced amino acid sequence of the L segment of this newly identified virus shows 57% sequence similarity (99% query coverage) with *Penicillium discovirus*. This virus also showed about 50% similarity to a few other tick viruses that were recently reported from China and the USA, such as Zhangzhou tick virus 1 (host: *Rhipicephalus sanguineus*), *Ixodes scapularis*-associated virus-5, and Guyuan tick virus 1 (host: *Haemaphysalis japonica*).

### 3.3. Double-Stranded RNA Viruses

#### 3.3.1. Partitiviridae

**Figure 6:**
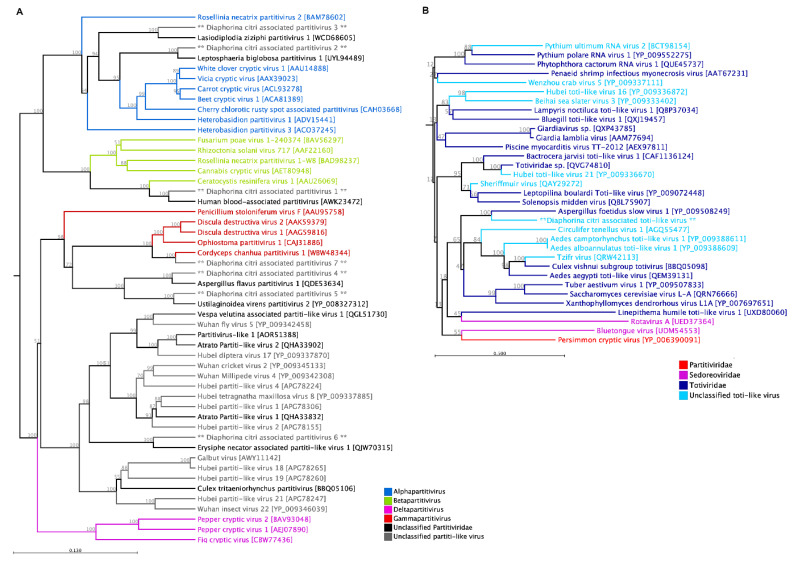
Phylogenetic relationships of putative double-stranded RNA viruses from the families *Partitiviridae* (**A**) and *Totiviridae* (**B**) detected in *D. citri*. Novel viruses identified in this study are marked with asterisks (**). The tree was constructed using the maximum-likelihood method based on MUSCLE-aligned amino acid sequences of predicted RdRP or ORF regions.

We identified seven bi-segmented partitiviruses associated with *D. citri*. They were tentatively named DcPV1 to DcPV7, and their newly identified sequences were deposited in the NCBI GenBank under the accession numbers of PV821400–PV821413 (Table 1). The *Partitiviridae* consists of small, isometric, non-enveloped viruses with bi-segmented dsRNA genomes ranging from 3 to 4.8 kbp. [50]. The largest partiti-like virus associated with *D. citri* is DcPV1 which has 2312 nt and 2196 nt in segments 1 and 2, respectively. Most of these novel viruses were identified in samples from northern Upolu. A Mononegavirales RdRp catalytic domain was detected on the first segment of DcPV1. Its deduced amino acid sequence showed high similarity to the coat protein of human blood-associated partitivirus and was classified under the *Betapartitivirus* clade by maximum-likelihood phylogenetic analysis. Two other Diaphorina citri-associated partitiviruses (DcPV2 and DcPV3) were found to share homology with two other fungal viruses, *Leptosphaeria biglobosa* partitivirus 1 and *Lasiodiplodia ziziphi* partitivirus 1, respectively, and are more likely members of the *Alphapartitivirus* genus based on their phylogenetic relationship with other known members of this genus (Figure 6A). DcPV7, which shares homology with *Cordyceps chanhua* partitivirus, grouped with other members of *Gammapartitivirus* genera. Other newly identified Diaphorina citri-associated partitiviruses made a distinct clade with many other unclassified members of *Partitiviridae*, which are newly reported form other arthropods and fungi. 

Within the *Partitiviridae*, there are five distinct genera, each exhibiting a preference for specific hosts: plants or fungi for the *Alphapartitivirus* and *Betapartitivirus* genera, fungi for the *Gammapartitivirus* genus, plants for the *Deltapartitivirus* genus, and protozoa for the *Cryspovirus* genus. The transmission of partitiviruses occurs intracellularly through various means, but no natural vectors have been identified for these viruses. Currently, there are 45 species assigned to the five partitivirus genera, with an additional 15 species that have yet to be assigned to a specific genus [50]. Similar to many other group viruses, metagenomic surveys have revealed that partitivirus-like sequences are also commonly associated with arthropods and other invertebrates. One arthropod-associated partitivirus, Galbut virus, is prevalent in wild populations of *Drosophila melanogaster*. Vertical transmission of this virus from either infected females or infected males is similar to Verdadero virus, another recently discovered partiti-like virus in a laboratory colony of *Aedes aegypti* mosquitoes [51]. Recently, three non-segmented parititi-like viruses were also identified in omnivorous insects such as the ant beetle (*Thanasimus formicarius*), the shore beetle (*Haliplus fluviatilis*), and a Collembola (*Tetrodontophora bielanensis*) [29]. It was previously thought that members of the *Partitiviridae* possessed two genomic segments. However, the identification of the capsid-encoded segment within these newly discovered insect-associated virus groups including DcPVs might change our understanding of this family. It appears that insects harbor a significantly diverse community of partitiviruses, which could potentially serve as primary reservoirs and/or transmission vectors for partitiviruses found in plants or fungi.

#### 3.3.2. Totiviridae

The *Totiviridae* are non-enveloped, double-stranded positive sense RNA viruses with icosahedral virions, which consist of a single capsid protein [52]. This is the first report of a toti-like virus in ACP, which we have tentatively named Diaphorina citri-associated toti-like virus (DcTv1). The complete genome sequence of this novel virus (GenBank accession number: PV821399) consists of two open reading frames within a single segment of 5039 bp (Figure 6B) with a 62.4% G + C content. The genome has 281 and 48 nucleotides in its 5′ and 3′ UTR, respectively. The first ORF is 2331 nt, produces 777 amino acids (aa), and shares 65.6% aa sequence identity (query coverage, 87%) with the coat protein of Erysiphe necator-associated totivirus 1, which was identified in Italy through a study of mycovirome associated with the plant pathogenic fungus *Erysiphe necator*. This ORF also showed 39.7% aa sequence similarity with 79% query coverage to Hangzhou totivirus 4 (UHK03338.1), which was isolated from zigzag leafhopper (*Recilia dorsalis*) in China (PRJNA629998). Luoyang Totiv tick virus 4 (UYL95684.1) isolated from the Asian longhorned tick *Haemaphysalis longicornis* in China had the maximum sequence coverage of 86% (sequence similarity of 58.1%) with the first ORF of this newly identified virus. The second ORF consists of 767 aa and showed the maximum shared sequence identity (59.6%) and coverage (97%) with RNA-dependent RNA polymerase of Erysiphe necator-associated totivirus 1 (QLC27590.1)

Traditionally, fungi and protozoa have been recognised as hosts for viruses from *Totiviridae*, but recent metagenomic analysis has revealed that they also infect much more advanced hosts. Several unclassified toti-like viruses have been identified from arthropods, fish, worms, and insectivorous bats [53,54,55], and the discovery of DcTv1 may provide further evidence to support their wider host range. However, the disease-causing potential and economic impact of these viruses in agriculture and public health is poorly described.

#### 3.3.3. Polymycoviridae

In this study, we identified the RNA4 segment of a novel double-stranded RNA virus (NCBI access code: PV821417). The RNA4 segment of polymycoviruses is a proline-alanine-serine protein-rich segment [56]. The virus sequence had a length of 1072 nt and a single open reading frame consisting of 318 aa. This segment sequence was obtained from all three location samples of *D. citri*. We putatively named it Diaphorina citri-associated polymycovirus (DcPMv) belonging to the *Polymycoviridae* family. Members of the *Polymycoviridae* family have a viral protein coat and can have either four or eight segments [56]. They have been isolated from various fungal hosts [57,58] and have yet to be identified from insects.

**Figure 7:**
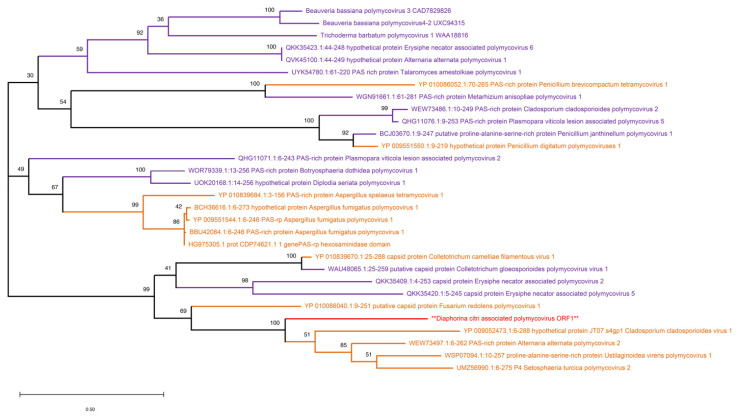
Maximum-likelihood phylogenetic tree of Diaphorina citri-associated polymycovirus (DcPMv). The tree is based on the amino acid sequence of the RNA4 segment encoding a PAS-rich protein. DcPMv clusters with fungal polymycoviruses, including Alternaria alternata polymycovirus 2, suggesting a fungal origin. The novel virus identified in this study is marked with an asterisk (**) in red color.

BLASTp results showed maximum alignment score and 56.03% shared identity with the PAS-rich protein region of *Alternaria alternata* polymycovirus 2 (WEW73497.1), which was isolated from grapevine trunk, but since it is a common pathogen of the *A. alternata* fungus, this newly discovered virus is likely to be of fungal origin. It also showed 55.65% shared identity with *Ustilaginoidea virens* polymycovirus 1 (WSP07094.1) and 50.92% shared identity with *Setosphaeria turcica* polymycovirus 2 (UMZ56990.1). Additionally, it showed 88% query coverage and 50.35% shared identity with *Cladosporium cladosporioides* virus 1 (YP_009052473.1). It also showed that DcPMv is grouping with these above viruses in the maximum likelihood tree (Figure 7). The lower read count in comparison with other identified novel viruses in this study coupled with the fact that polymycoviruses have primarily fungal hosts indicates that DcPMv is a fungal virus rather than an insect-specific virus.

### 3.4. Microbiota Diversity and Screening for the CLas

We previously screened 287 *D. citri* specimens collected in 2019 for *C*Las. No PCR amplification was observed in any of the samples, indicating the likely absence of the pathogen in the tested population. The positive control confirmed that the PCR assay was successful, ruling out the possibility of technical failure (Figure S2). However, false negative rates are considered high, as the probability of detecting the bacterium can vary depending on the time of year and the physiological age of the host plant, which may influence pathogen titre and detectability [59,60]. Screening a large number of individuals and conducting sequential surveillance throughout the year are therefore recommended [59]. Therefore, although all screened samples showed no evidence of being infected by the bacteria, we cannot entirely confirm that it is absent from Samoa. Further screening would be prudent with a focus on obtaining samples from plants that are more likely to be infected.

**Figure 8:**
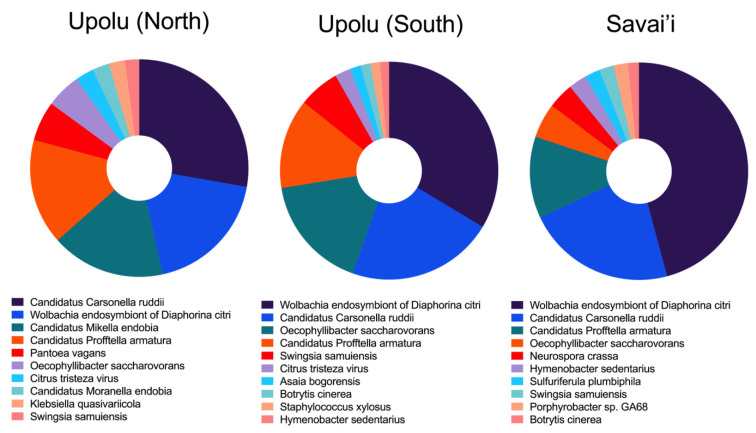
Microbiota composition of *Diaphorina citri* populations from three Samoan locations. Pie charts represent the relative abundance of dominant bacterial taxa identified in *D. citri* individuals collected from Upolu North, Upolu South, and Savai’i.

To assess the presence or absence of *C*Las in the Samoan population of *D. citri*, we also characterised the microbiota composition of the sequenced samples using Kraken2. The microbiome profiles were found to be highly consistent across all three sampling locations, indicating a stable core microbial community within this population (Figure 8). This analysis serves as a preliminary catalogue of putative bacterial taxa, and any interpretations regarding their presence should be treated with caution until validated through targeted wet lab experiments. *D. citri* harbors a diverse assemblage of microorganisms that play critical roles in its physiology, fitness, and capacity to transmit pathogens such as *C*Las. This community includes both bacterial endosymbionts and fungal associates, each of which may influence the psyllid’s survival, reproductive success, and vector competence.

The most abundant identified taxa include Ca. *Carsonella ruddii*, *Wolbachia* endosymbiont of *D. citri*, Ca. *Profftella armatura*, and several other species contributing to the microbiota diversity in these populations (Figure 8 and Supplementary Table S1). Among them, the phylum Proteobacteria was identified as a dominant group of bacteria with 73.3% of the abundance, which is recognised for their diversity and significance in a variety of ecological roles, including symbiotic relationships with arthropods. The primary endosymbiont *Wolbachia*, belonging to the Alphasproteobacteria class, a common symbiotic bacterium frequently identified in arthropod vectors, was the most prevalent bacterium among them. *Wolbachia* is known to exert various effects on its hosts, most notably the capacity to control reproduction through processes like cytoplasmic incompatibility (CI). CI helps in maternal transmission of the bacteria, in which only females infected with a particular *Wolbachia* strain can successfully produce embryos with both infected and uninfected males [61].

Previous studies showed that the *Wolbachia* in the larvae of the *Phyllonorycter blancardella* can promote the secretion of cytokinins from leaves, delay leaf senescence, and increase the host’s feeding time on leaves [62]. The improvement of this adaptability is of great significance for insect hosts to survive and reproduce in complex and changing ecological environments. Symbiotic microorganisms can also affect the transmission of pathogenic microorganisms by vector insects, affecting the growth and development of plants and the process of pathogen transmission. Studies have shown that the transmission efficiency of Bergomovirus by tobacco whiteflies infected with the endosymbiotic microorganism *Wolbachia* is improved [63].

Additionally, two species from the *Candidatus* genus were identified, *C. Carsonella ruddii* and *C. Profftella armatura*. A combination of these species has been previously reported from psyllids, and they are known endosymbionts of *D. citri* [64,65]. These bacteria contributed to 10–12% and 5–7% of the total bacterial reads, respectively. In particular, *C. Carsonella ruddii* is involved in the biosynthesis of essential nutrients that the insect host cannot obtain from its diet, while *C. Profftella armatura* has been associated with the production of defensive compounds like toxins that protect the holobiont [66]. The bacterium *Oecophyllibacter saccharovorans* exhibited a high read count, particularly in the UPO2 sample from southern Upolu, with over 100,000 reads. This bacterium has been previously associated with the weaver ant, *Oecophylla smaragdina* [67]. The high abundance of *O. saccharovorans* in *D. citri* at this location suggests possible site-specific environmental factors or interactions with other organisms that promote its proliferation.

Although no *C*Las positive *D. citri* individuals were detected using conventional PCR, Kraken2 analysis revealed low read counts (approximately 8–16) assigned to *C*Las across the samples. Given the high total read depth per sample, these low-abundance hits are likely to represent background noise or spurious taxonomic assignments. Moreover, these reads did not align to genomic regions uniquely specific to *C*Las, further suggesting the possibility of misclassification rather than true presence (Supplementary Table S1).

Beyond bacterial symbionts, fungal organisms were also identified across all three sampling locations. Among these, *Botrytis cinerea*—a well-known plant pathogen responsible for grey mould disease—was detected specifically in samples from South Upolu (UPO2) and Savai’I (SAV1). The presence of *B. cinerea* in the microbiome of *D citri* suggests a potential ecological interaction involving the psyllid, the fungus, and the host plants. While the nature of this association remains uncertain, it may reflect environmental exposure, incidental ingestion, or a more complex tripartite relationship.

During our virome analysis of *D. citri*, we identified several viral sequences that are more likely to infect fungal hosts than insects. The concurrent detection of diverse fungal taxa in the *D. citri* microbiome supports the presence of mycoviruses within these samples. Notably, *Neurospora crassa*, a well-established model organism for studying mycovirus replication [68], was detected at the SAV1 site, comprising 1.84% of the total reads. These findings suggest that some of the newly identified viruses may be associated with fungal species residing in the *D. citri* gut, rather than the insect itself. Further investigation using small RNA sequencing could help elucidate the complex interactions between these viruses and their potential fungal hosts.

Overall, our results highlight the potential for both fungi and their associated viruses to influence the structure and function of the *D. citri* microbiome, with possible implications for the insect’s health, ecology, and vectoring capacity. Further research could involve culturing the identified endosymbionts in the laboratory and performing functional assays to determine their roles in the biology of *D. citri*. This might be further explored by using antibiotic treatments to selectively eliminate specific bacteria and evaluate the resulting effects on the psyllid host’s survival, as well as *D. citri* reproduction and its ability to transmit plant pathogens. Additionally, studying the mechanisms of vertical transmission would provide insights into how these symbionts are maintained across generations, potentially influencing the evolutionary success of the insect host. Understanding the complex interplay between bacteria, fungi, and viruses within the *D. citri* microbiome could also lead to new strategies for controlling this pest. This study of *D. citri*’s microbiome provides a better understanding of the symbiotic relationships that may contribute to the psyllid’s adaptability and effectiveness as a pest, with important implications for pest management strategies in agriculture.

## 4. Conclusions

The use of insect-specific viruses as biocontrol agents represents a promising and increasingly explored strategy for managing vector-borne plant diseases. Advances in high-throughput sequencing technologies have significantly enhanced our ability to detect and characterize novel viruses; however, our understanding of the geographical viromes of *D. citri* populations remains limited. Despite the ecological and economic importance of *D. citri* as a vector of *C*Las, only a small number of viruses have been thoroughly characterised in this species, and the complexity of their interactions with the insect host and associated plant pathogens is still poorly understood.

Previous studies have identified several viral families in *D. citri*, including picorna-like, flavi-like, mymonaviruses, and reoviruses, confirming the presence of insect-specific viruses. In contrast, our study did not detect many of these previously reported viruses. Instead, we identified several novel viruses associated with *D. citri* populations in Samoa, including partitivirus-like and bunyavirus-like viruses not previously reported in this vector. Phylogenetic analysis revealed that these newly identified viruses belong to a diverse range of viral families, including *Hypoviridae*, *Yadokariviridae*, *Botourmiaviridae*, *Nodaviridae*, *Narnaviridae*, *Discoviridae*, *Partitiviridae*, *Totiviridae*, *Polymycoviridae*, and *Splipalmiviridae*.

Our study expands the known virome diversity of *D. citri* and highlights potential regional variation in viral communities. However, significant knowledge gaps remain regarding the functional roles of these viruses, their potential for manipulating vector biology, and their broader implications for disease transmission dynamics.

## Figures and Tables

**Table 1:** Summary of novel viruses associated with *Diaphorina citri*. Each virus is listed with its closest related virus based on BLAST analysis and the highest percentage identity at both the protein (nr) and nucleotide (nt) levels.

Diaphorina citri-Associated Virus Name	Closely Related Virus	Accession Code	Greatest Identity % Protein Level (nr)	Greatest Identity % Nucleotide Level (nt)
hypovirus 1	Chuzhou tick virus 1	UYL95331	87.32	88.37
yadokarivirus 1	Aspergillus homomorphus yadokarivirus 1	AZT88626	58.67	77.20
mononega-like virus 1	Leptosphaeria biglobosa ss(-)RNA virus 4	UYL94505	57.74	76.69
toti-like virus	Erysiphe necator-associated totivirus 1	QLC27591	66.83	92.5
narnavirus 1	Rhizoctonia solani narnavirus 13	UIW13880	47.05	-
narnavirus 2	Plasmopara viticola lesion-associated narnavirus 4	QIR30283	75.18	92.50
narnavirus 3	Plasmopara viticola lesion-associated narnavirus 27	QIR30306	52.60	81.42
narnavirus 4	Botrytis cinerea binarnavirus 2	QLF49184	49.25	97.36
narnavirus 5	Erysiphe necator-associated narnavirus 29	QJT93761	55.64	84.31
narnavirus 6	Plasmopara viticola lesion-associated narnavirus 12	QIR30291	67.76	86.79
narnavirus 7	Sclerotinia sclerotiorum narnavirus 4	QZE12025	40.39	90.00
narnavirus 8	Tonghua Narna tick virus 3	UYL95393	55.16	83.13
narnavirus 9	Aspergillus creber narnavirus 1	BDB16249	32.00	-
narnavirus 10	Sclerotinia sclerotiorum narnavirus 1	QZE12035	40.09	97.29
splipalmivirus segment RNA1	Cryphonectria naterciae splipalmivirus 1	BCX55509	68.20	89.74
splipalmivirus segment RNA2	Cryphonectria naterciae splipalmivirus 1	BCX55510	66.71	75.90
splipalmivirus segment RNA3	Cryphonectria naterciae splipalmivirus 1	BCX55511	60.78	85.71
splipalmivirus segment RNA4	Oidiodendron maius splipalmivirus 1	UJT31791	44.26	74.25
partitivirus 1 Segment 1	Human blood-associated partitivirus	AWK23473	65.43	91.11
partitivirus 1 Segment 2	Human blood-associated partitivirus	AWK23472	75.70	86.79
partitivirus 2 Segment 1	Leptosphaeria biglobosa partitivirus 1	UYL94490	63.34	75.25
partitivirus 2 Segment 2	Leptosphaeria biglobosa partitivirus 1	UYL94489	83.04	90.69
partitivirus 3 Segment 1	Lichen partiti-like RNA virus 2	BCD56386	53.13	69.48
partitivirus 3 Segment 2	Lasiodiplodia ziziphi partitivirus 1	WCD68605	70.66	88.37
partitivirus 4 Segment 1	Aspergillus flavus partitivirus 1	BED98277	45.16	-
partitivirus 4 Segment 2	Aspergillus flavus partitivirus 1	QDE53634	80.90	79.66
partitivirus 5 Segment 1	Ustilaginoidea virens partitivirus 2	YP_008327313	71.77	80.55
partitivirus 5 Segment 2	Ustilaginoidea virens partitivirus 2	YP_008327312	79.01	83.82
partitivirus 6 Segment 1	Metarhizium brunneum partitivirus 1	QHB49874	56.74	79.66
partitivirus 6 Segment 2	Erysiphe necator-associated partiti-like virus 1	QJW70315	67.69	80.32
partitivirus 7 Segment 1	Cordyceps chanhua partitivirus 1	WBW48345	69.03	88.00
partitivirus 7 Segment 2	Cordyceps chanhua partitivirus 1	WBW48344	83.91	90.24
virga-like virus 1	Penicillium vanoranjei-associated RNA virus 1	BDF97667	87.01	82.49
discovirus L	Peribunyaviridae sp.	WAK75613	64.78	80.28
discovirus M	Penicillium discovirus	YP_010840286	44.06	-
discovirus S	Penicillium discovirus	YP_010840287	53.50	-
polymycovirus segment RNA4	Alternaria alternata polymycovirus 2	WEW73497	56.69	75.23
ourmia-like virus 1	Tianjin Botou tick virus 4	UYL95459	74.538	94.28
ourmia-like virus 2	*Narnaviridae* sp.	WAK75260	74.00	96.87

A dash (-) indicates that no significant nucleotide identity was found or reported.

**Table 2:** Genomic features, abundance, and protein domain annotations of novel viruses associated with *Diaphorina citri* in Samoa.

Diaphorina citri-Associated Virus Name	Virus Length	Assembly Coverage	Total Read Count			InterPro Accession	InterPro Name
			Upolo N	Upolo S	Savai’i		
ss(+)RNA viruses							
hypovirus 1 (DcHv1)	11431	97.57	7464	1	1	IPR027417; IPR021912; IPR014001; IPR043502	DNA/RNA polymerase superfamily; P-loop containing nucleoside triphosphate hydrolase; protein of unknown function DUF3525; Helicase superfamily 1/2 (ATP-binding domain)
yadokarivirus 1 (DcYv1)	3828	612.69	15,746	2	1	IPR001205; IPR043502	RNA-directed RNA polymerase, C-terminal domain; DNA/RNA polymerase
ourmia-like virus 1 (DcOv1)	1739	6.15	8	7	58	IPR043502	DNA/RNA polymerase superfamily
ourmia-like virus 2 (DcOv2)	1370	6.95	12	5	52	-	-
virga-like virus 1 (DcV1)	1685	11.99	2	8	126	IPR001788; IPR007094; IPR043502	RNA-directed RNA polymerase, C-terminal domain; RNA-directed RNA polymerase, catalytic domain; DNA/RNA polymerase superfamily
narnavirus 1 (DcNv1)	2383	505.47	8066	1	0	IPR008686; IPR043502	RNA-dependent RNA polymerase, mitoviral; DNA/RNA polymerase superfamily
narnavirus 2 (DcNv2)	2524	893.83	15,101	2	2	-	-
narnavirus 3 (DcNv3)	3552	1126.52	33,473	12,073	9510	-	-
narnavirus 4 (DcNv4)	2260	837.95	12,676	7	3	IPR008871	Totivirus coat
narnavirus 5 (DcNv5)	2260	9.96	150	2	0	-	-
narnavirus 6 (DcNv6)	3409	583.8	13,334	1	2	-	-
narnavirus 7 (DcNv7)	2477	1439.67	23,844	0	1	IPR043502	DNA/RNA polymerase superfamily
narnavirus 8 (DcNv8)	2351	13.87	96	3	127	-	-
narnavirus 9 (DcNv9)	1584	1123.06	15,863	7626	6146	-	-
narnavirus 10 (DcNv10)	941	1738.49	10,941	0	0	IPR007099	RNA-directed RNA polymerase, negative-strand RNA virus
splipalmivirus segment RNA1 (DcSV1)	2164	94.25	1370	1	0	IPR008871	Totivirus coat
splipalmivirus segment RNA2 (DcSV2)	2273	76.63	1166	1	0	IPR043502	DNA/RNA polymerase superfamily
splipalmivirus segment RNA3 (DcSV3)	1459	575.82	5619	0	0	-	-
splipalmivirus segment RNA4 (DcSV4)	533	2.85	11	0	0	-	-
**ss(-)RNA viruses**							
mymona-like virus 1 (DcMv1)	9708	92.17	5353	3116	1261	IPR026890; IPR014023	Mononegavirales RNA-directed RNA polymerase catalytic domain; mononegavirales mRNA-capping domain V
discovirus L (DcDv-L)	6556	62.4	2739	0	0	IPR007322; IPR007099	RNA-dependent RNA polymerase, bunyaviral; RNA-directed RNA polymerase, negative-strand RNA virus
discovirus M (DcDv-M)	1221	675.19	5513	0	0	-	-
discovirus S (DcDv-S)	1054	464.71	3277	2	1	-	-
**dsRNA viruses**							
partitivirus 1 Segment 1 (DcPv1-S1)	2312	146.82	2271	5	1	IPR026890;	Mononegavirales RNA-directed RNA polymerase catalytic domain
partitivirus 1 Segment 2 (DcPv1-S2)	2196	138.24	2032	4	3	IPR001205; IPR043502	RNA-directed RNA polymerase, C-terminal domain; DNA/RNA polymerase superfamily
partitivirus 2 Segment 1 (DcPv2-S1)	1808	27.67	493	196	118	IPR043502	DNA/RNA polymerase superfamily
partitivirus 2 Segment 2 (DcPv2-S2)	1917	120.57	1549	8	11	IPR001205; IPR043502	RNA-directed RNA polymerase, C-terminal domain; DNA/RNA polymerase superfamily
partitivirus 3 Segment 1 (DcPv3-S1)	1732	61.73	716	0	0	IPR008871	Totivirus coat
partitivirus 3 Segment 2 (DcPv3-S2)	1902	140.39	1788	0	0	IPR001205; IPR043502	RNA-directed RNA polymerase, C-terminal domain; DNA/RNA polymerase superfamily
partitivirus 4 Segment 1 (DcPv4-S1)	1331	62.36	558	3	2	-	-
partitivirus 4 Segment 2 (DcPv4-S2)	1735	156.1	1811	0	1	IPR001205; IPR007094; IPR043502	RNA-directed RNA polymerase, C-terminal domain; RNA-directed RNA polymerase, catalytic domain; DNA/RNA polymerase superfamily
partitivirus 5 Segment 1 (DcPv5-S1)	1329	168.77	1503	0	0	-	-
partitivirus 5 Segment 2 (DcPv5-S2)	1736	387.13	4502	1	0	IPR001205; IPR007094; IPR043502	RNA-directed RNA polymerase, C-terminal domain; RNA-directed RNA polymerase, catalytic domain; DNA/RNA polymerase superfamily
partitivirus 6 Segment 1 (DcPv6-S1)	1712	115	1317	2	1	IPR008871	Totivirus coat
partitivirus 6 Segment 2 (DcPv6-S2)	1813	103.77	1261	0	0	IPR001205; IPR007094; IPR043502	RNA-directed RNA polymerase, C-terminal domain; RNA-directed RNA polymerase, catalytic domain; DNA/RNA polymerase superfamily
partitivirus 7 Segment 1 (DcPv7-S1)	1581	75.97	803	1	0	IPR043502	DNA/RNA polymerase superfamily
partitivirus 7 Segment 2 (DcPv7-S2)	1752	108.23	1271	1	0	IPR043128; IPR001205; IPR007094; IPR043502	Reverse transcriptase/Diguanylate cyclase domain; RNA-directed RNA polymerase, C-terminal domain; RNA-directed RNA polymerase, catalytic domain; DNA/RNA polymerase superfamily
toti-like virus (DcTv)	5039	38.36	1294	1	1	IPR008871	Totivirus coat
polymycovirus segment RNA4 (DcPMv)	1072	33.24	323	106	83	IPR043502	DNA/RNA polymerase superfamily

## Data Availability

Deep sequencing raw data were deposited in the National Centre for Biotechnology Information’s (NCBI’s) Sequence Read Archive (SRA) and are accessible through BioProject series accession number PRJNA1275559.

## Supplementary Materials

The following supporting information can be downloaded at: https://www.mdpi.com/article/10.3390/pathogens14080801/s1, Figure S1: Map of the Samoan Islands showing Upolu (A) and Savai’i (B), with marked sampling sites and survey locations.; Figure S2: PCR detection of *Ca.* Liberibacter spp. in Asian citrus psyllid samples. Table S1: Taxonomic composition of sequences detected in three samples based on Kraken 2 analysis. Counts represent the number of sequence reads assigned to each taxonomic group in each sample..
