# Supplementary figures and images for: Characterising the Associated Virome and Microbiota of Asian Citrus Psyllid (Diaphorina citri) in Samoa
Source: Pathogens. 2025 Aug 10;14(8):801. doi: 10.3390/pathogens14080801 (PMC12389512; doi:10.3390/pathogens14080801)

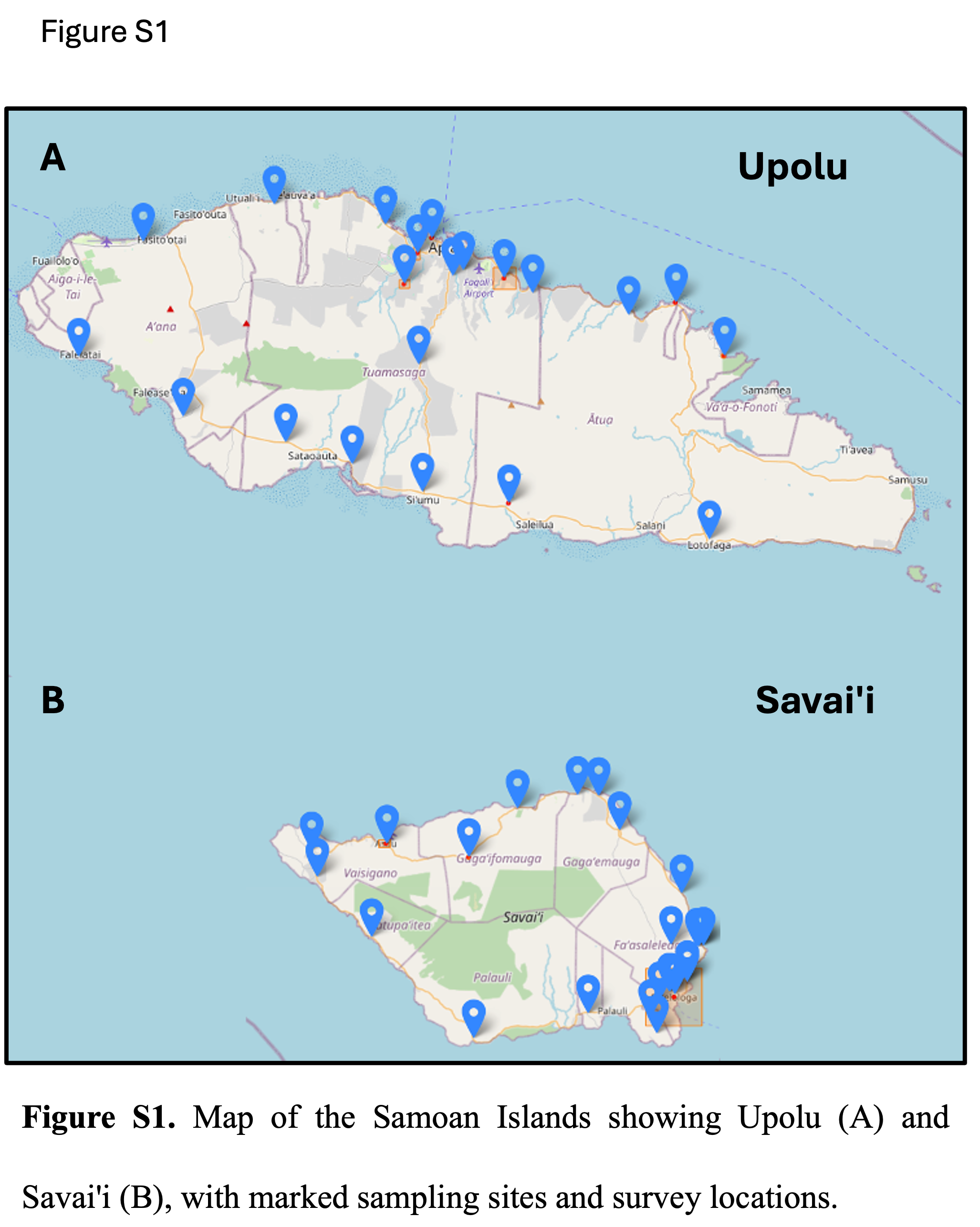

Supplement: Supplementary file 1 [file pathogens-14-00801-s001.zip › Figure S1.tif]

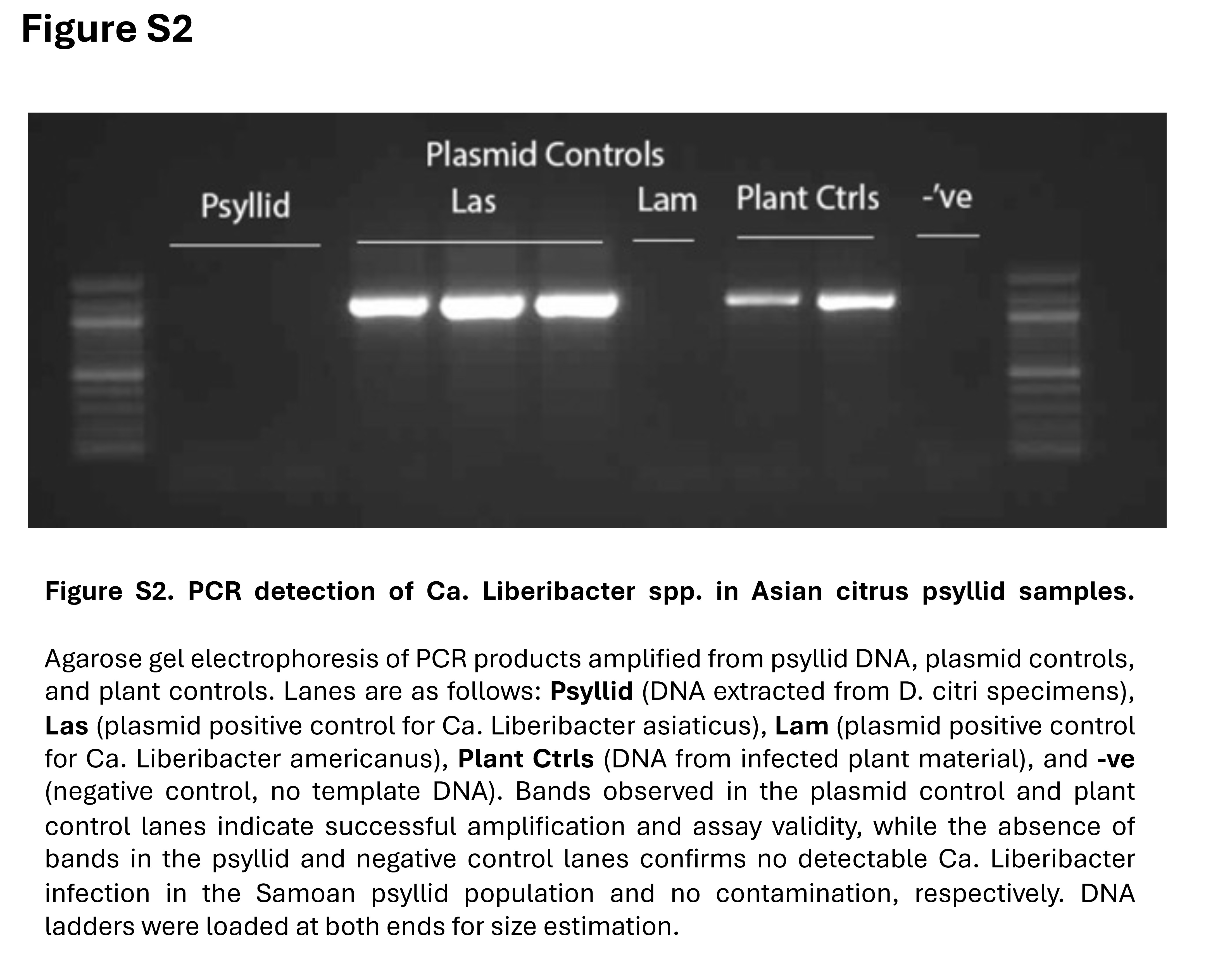

Supplement: Supplementary file 1 [file pathogens-14-00801-s001.zip › Figure S2.tif]
